# Acculturation Profiles of Weight Perception Status among US Foreign-Born Hispanic/Latino Adults: A Mixture Model Approach

**DOI:** 10.3390/ijerph19159704

**Published:** 2022-08-06

**Authors:** Kevin Villalobos, Francisco A. Montiel Ishino, Faustine Williams

**Affiliations:** 1 Division of Intramural Research, National Institute on Minority Health and Health Disparities, Bethesda, MD 20892, USA; 2 National Institutes of Health, Bethesda, MD 20892, USA

**Keywords:** acculturation, Hispanic/Latino, weight perception, health disparities

## Abstract

The objective of this study was to identify profiles of acculturation and weight-by-weight perception status among United States (US) foreign-born Hispanic/Latino adults using a person-centered approach. We conducted a latent class analysis (LCA) on 1999–2004 National Health and Nutrition Examination Survey (NHANES) data from US foreign-born Hispanic/Latino adults 18 years and older (N = 4944). Acculturation was assessed by self-reported linguistic acculturation questions from the Short Acculturation Scale for Hispanics. Weight was assessed by body mass index (BMI). Covariates included weight perception and sociodemographic factors to compare and further differentiate profiles. Three profiles were identified: bicultural (15% of sample), low acculturation (84%), and non-integrated (1%). All the profiles had a BMI that was considered overweight or obese. The low acculturated profile was less likely (odds ratio (OR): 0.62, 95% confidence interval (CI): 0.43–0.91) to perceive themselves as overweight relative to the bicultural class. The low accultured profile was also more likely to be female and a US citizen (OR: 1.45, 95% CI: 1.09–1.92 and OR: 2.29, 95% CI: 1.57–3.34) in comparison to the bicultural class. Our study is among the first to use LCA to examine weight perception on acculturation status and weight profiles among US foreign-born Hispanic/Latino adults. The findings of our study are a step towards building a foundation to mitigate weight disparities among underserved/underrepresented US foreign-born individuals, especially Hispanics/Latinos. Our results can also inform the development of tailored interventions.

## 1. Introduction

Overweight and obesity is an alarming epidemic that affects millions of individuals living in the United States (US). The obesity prevalence among US adults in 2018 was approximately 42.4% [1]. Complications related to obesity are type 2 diabetes, heart disease, dyslipidemia, and mental health [1,2,3]. Overweight and obesity has also been attributed to decreased life expectancy and increase mortality rate [4,5]. According to the Centers for Disease Control and Prevention [1], this disparity affects racial/ethnic populations at different rates; the 2018 age-adjusted prevalence of obesity was the second highest among Hispanic adults (44.8%). There is a public health need to understand risk in the context of acculturation among US Hispanics/Latinos, especially among the foreign-born subpopulations, as they are the fastest growing group in the country. The relationship between acculturation and obesity among migrant populations has been reported to increase the more acculturated they become [6]. Yet not much is known about the role of weight perception on this relationship. As such, the effect of acculturation in the overweight/obesity epidemic among Hispanics/Latinos must be explored.

Using nationally representative data, Guendelman et al. [7], conducted a US–Mexico binational analysis for weight perception among Mexican and Mexican-American women living with overweight or obesity. This study found that Mexican women were less likely to correctly label themselves as overweight when compared to Mexican-American women [7]. Regarding Mexican-American men, Lewis et al. [8] observed that physical characteristics were associated with individuals living with overweight or obesity. Specifically, Mexican-American men were more likely to underestimate their weight when compared to White and Black participants [8].

Several studies have indicated that Hispanics/Latinos are experiencing a synergistic overweight/obesity epidemic which is ultimately affecting their overall health [9,10,11]. Moreover, it has been reported that the overweight/obesity epidemic effects are especially prevalent among Hispanic/Latino women in the US [12]. The purpose of this study was to identify subgroups of US foreign-born Hispanics/Latinos adults based on acculturation and body mass index (BMI), as well as further differentiating profiles based on weight perception and sociodemographic characteristics. While previous studies have analyzed the association between Hispanics/Latinos and weight perception, our latent class person-centered approach identified subgroups using complex survey design data to fill a critical gap in the literature concerning US Hispanic/Latino disparities.

## 2. Materials and Methods

Latent class analysis (LCA)—a mixture model analytic approach used to identify hidden or latent groups from observed indicators [13]—was used on 1999–2004 National Health and Nutrition Examination Survey (NHANES) data of adults 18 and older that had acculturation and BMI data (N = 4944). All data were analyzed using weights, strata, and clusters to appropriately account for the complex survey design. All data are publicly available from the Centers for Disease Control and Prevention—National Center for Health Statistics (https://wwwn.cdc.gov/nchs/nhanes/default.aspx, accessed on 4 March 2022). No Institutional Review Board review was necessary as no human subjects were involved in our study.

### 2.1. Latent Class Analysis

Body mass index was used as a continuous observed variable. Body mass index was collected and processed by the NHANES using participants’ weight in kilograms, which was then divided by their height in meters squared. Acculturation latent class was constructed from language use and years spent living in the US. Language use was based on linguistic acculturation questions from the Short Acculturation Scale for Hispanics and included language(s) used (1) with friends; (2) to think; (3) at home; (4) as a child; and (5) to read and speak. Languages were categorized as only/mostly Spanish (only Spanish; more Spanish than English), Spanish and English equally, and majority English (only English; more English than Spanish). Time living in the US was categorized as (1) <1 year; (2) 1 to <5 years; (3) 5 to <10 years; and (4) ≥10 years.

Covariates for auxiliary multinomial logistic regression for latent class differentiation included weight perception (self-perceived normal and underweight as reference compared to perceived as overweight) and sociodemographic factors. Sociodemographic factors included sex/gender (male as reference compared to female) and US citizenship (not a US citizen as reference compared to US citizen).

### 2.2. Analysis Plan

We used a comparative model fit approach to determine the number of classes to be interpreted. Models were created from 1- to 7-class solutions to then select the best model using the following criteria: (1) Bayesian information criterion (BIC); (2) sample-size-adjusted (ssa) BIC; (3) entropy or acceptable quality of classification; and (4) theoretical and practical implications [13]. An auxiliary multivariate logistic regression was used on covariates that included weight perception, sex/gender, and US citizenship to better define class memberships for the model selected for interpretation. All LCAs were conducted using Mplus version 8.5 (Muthén and Muthén). All code used in our analytical procedure will be made available by reasonable request.

## 3. Results

The foreign-born Hispanic/Latino sample was about evenly male (50.2%) and female (49.8%), non-US citizen (67.4%), lived in the US for ≥10 years (58.9%), and did not perceive themselves overweight (58.1%). They primarily only/mostly spoke Spanish with friends (67.1%), only/mostly used Spanish to think (72.4%), only/mostly used Spanish at home (77.7%), only/mostly used Spanish as a child (92.4%), and only/mostly used Spanish to read and speak (73.9%). See Table 1 for further detail.

### 3.1. Latent Class Analysis Findings

**Figure 1:**
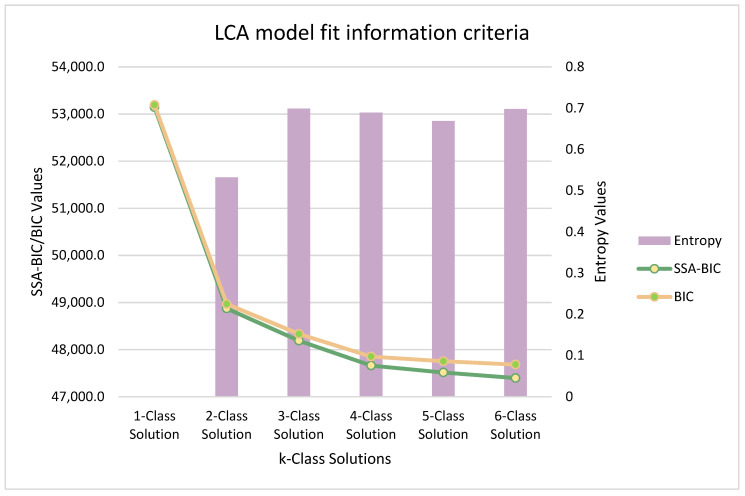
Fit model criteria.

The three-class solution was selected for interpretation (see Figure 1). Profiles were named based on linguistic acculturation categories: (Class 1) bicultural, (Class 2) low acculturated, and (Class 3) non-integrated.

Class 1, or the bicultural profile (15% of sample), had an intermediate mean BMI of 26.1 when compared to all the classes. This class had the highest conditional probability for time living in the US: ≥10 years (77.6%). Class 1 had the highest conditional probabilities for language used with friends: Spanish and English equally (47.9%), language used to think: Spanish and English equally (47.4%), language used at home: Spanish and English equally (35.3%), language read and spoken: Spanish and English equally (56.2%). This class also had a high conditional probability for language used as a child: only/mostly Spanish (77.5%).

Class 2, or the low acculturated profile (84% of sample), had the lowest mean BMI, 25.8, when compared to all the classes. This class had the highest conditional probabilities for language used with friends: only/mostly Spanish (94.0%), language used to think: only/mostly Spanish (97.6%), language used at home: only/mostly Spanish (98.4%), language used as a child: only/mostly Spanish (99.5%), language read and spoken: only/mostly Spanish (98.3%). This class also had a high conditional probability for time living in the US: ≥10 years (50.2%).

Class 3, or the non-integrated profile (1% of sample), had the highest mean BMI, 51.7, when compared to all the classes. This class had the highest conditional probabilities for language used with friends: only/mostly Spanish (88.2%), language used to think: only/mostly Spanish (87.3%), language used at home: only/mostly Spanish (89.7%), language used as a child: only/mostly Spanish (89.7%), language read and spoken: only/mostly Spanish (84.8%). This class also had a high conditional probability for time living in the US: ≥10 years (61.5%). See Table 2 for further detail.

### 3.2. Covariates

Our covariate analysis using multinomial logistic regression revealed the following. In consideration of sociodemographic factors, we found that persons had increased odds of being female (odds ratio (OR): 1.45, 95% confidence interval (CI): 1.09–1.92) in the low acculturated profile (Class 2) compared to the bicultural profile (Class 1). In addition to having increased odds of being female, the low acculturated profile (Class 2) had increased odds of having US citizenship (OR: 2.29, 95% CI: 1.57–3.34) compared to the bicultural profile (Class 1). The low acculturated profile (Class 2) had decreased odds of perceiving themselves as overweight (OR: 0.62, 95% CI: 0.43–0.91) when compared to the bicultural profile (Class 1). See Table 3 for further detail.

## 4. Discussion

We used latent class analysis, a person-centered approach, to identify heterogenous profiles among foreign-born Hispanic/Latino adults based on acculturation and BMI. Our analysis yielded three profiles, bicultural (Class 1), low acculturated (Class 2), and non-integrated (Class 3). The bicultural profile had the highest conditional probabilities for language read and spoken as Spanish and English equally and living in the US ≥10 years, with a BMI of 26.1.

The low acculturated profile had the highest conditional probabilities for only and mostly Spanish language used with friends, to think, at home, used as a child, and language read and spoken, as well as the lowest mean BMI of 25.8, when compared to the other profiles. The persons in this profile were more likely to be female and not US citizens. They were also less likely to perceive themselves as overweight. This result is consistent with Ahluwalia et al. [14], who analyzed NHANES from 2001–2002 and found that less acculturated Mexican-Americans with BMI ≥ 25 were less likely to perceive themselves as overweight and had 51% less odds to try to lose weight in the past 12 months (OR: 0.49, 95% CI: 0.31–0.79) relative to the more acculturated. Health promotion strategies are critical to addressing this weight issue, especially as this subgroup perceives themselves to be underweight although they are at the cusp of being overweight. Most importantly, this behavior could ultimately increase the risk of developing diabetes and cardiovascular disease. A study that focused on acculturation and the prevalence of diabetes in US Latino adults found an association between acculturation and increased diabetes risk [15]. Similarly, a study that focused on the prevalence of major cardiovascular risk factors and cardiovascular disease among Hispanics/Latinos in the US found that those that had higher levels of acculturation experienced an increased prevalence of cardiovascular disease risk [16]. Future research should examine the dimensions of bicultural acculturation and its effect on weight perception among US foreign-born Hispanics/Latinos.

The non-integrated profile was marked by an exclusive absence of Spanish and English being spoken equally in any language use category, with the highest mean BMI of 51.7 compared to the other profiles. This profile was marked by the second-highest conditional probabilities of only/mostly Spanish language and only/mostly English language used with friends, to think, at home, used as a child, and language read and spoken. The non-integrated profile was unique, as they had the highest risk of living with obesity and only or mostly spoke one language. This subgroup has lived in the US for ≥10 years but may not be adapting linguistically to the US or rejecting one culture linguistically by accepting or rejecting their Hispanic/Latino heritage. Future studies should focus on how sociocultural factors, nationality, acculturation, and time living in the US affect physical and mental health. Additional studies should also target the development of dynamic acculturation questionnaires that allow for a multidimensional comprehension of the issue.

Some limitations must be considered. First is that BMI may be a controversial measure for overweight and obesity given the level of variation in non-White racial and ethnic groups [17,18]. Nonetheless, the NHANES collects BMI measures in a clinical setting to minimize data collection error and measurement bias [19]. The second limitation is that acculturation, while a multidimensional process, was only measured using language and cross-sectionally in the NHANES. Multiple factors must be considered in acculturation, but the limitations of nationally representative survey data outweigh the limitations in helping generate hypotheses and develop future contextual studies. Lastly, this was secondary data analysis using cross-sectional data with which we could not capture the temporal nature of acculturation. However, our study was not without strengths, as we used a nationally representative sample of US Hispanics/Latinos across multiple survey years to identify hidden profiles from observed acculturation indicators. Our findings help reveal the most at-risk among the already at-risk to help develop tailored intervention programs, as well as explore the identified risk profiles in community settings.

## 5. Conclusions

We conducted a latent class analysis, a person-centered approach, to identify unobserved profiles. Our analysis identified three profiles: bicultural profile, low acculturated profile, and non-integrated profile. Findings indicate that all profiles were considered overweight or obese. We also found that persons in the low acculturated profile were less likely to perceive themselves as overweight. It is important to acknowledge that we identified a profile that had a mean BMI of 51.7. Ultimately, our analysis allowed for the identification of profiles that are potentially at most risk. The results from this study can be used as evidence to strengthen the ongoing approach to understanding/preventing obesity syndemic, serve as leverage for complex survey designs, and further research to promote the development of tailored interventions for US foreign-born Hispanics/Latinos.

## Figures and Tables

**Table 1:** Sample statistics (N = 4944).

	N	%
**Language used with friends**		
Only/Mostly Spanish	1613	67.1
Spanish and English Equally	443	18.4
Only/Mostly English	346	14.4
**Language used to think**		
Only/Mostly Spanish	1733	72.4
Spanish and English Equally	391	16.3
Only/Mostly English	270	11.3
**Language used at home**		
Only/Mostly Spanish	1866	77.7
Spanish and English Equally	284	11.8
Only/Mostly English	252	10.5
**Language used as a child**		
Only/Mostly Spanish	2218	92.4
Spanish and English Equally	134	5.6
Only/Mostly English	49	2
**Language read and spoken**		
Only/Mostly Spanish	1775	73.9
Spanish and English Equally	455	19
Only/Mostly English	172	7.2
**Time living in the US**		
Less than a year	321	6.6
1 to 4.9 years	866	17.8
5 to 9.9 years	812	16.7
10 or more years	2870	58.9
**Gender**		
Male	2485	50.2
Female	2469	49.8
**Overweight Perception**		
Not Perceived	2384	50.2
Perceived	1717	49.8
**Citizenship**		
US Citizenship	1600	32.6
Non-US Citizenship	3303	67.4
	**Mean**	**SE**
**Body Mass Index**	26.2	2.21

Notes: SE = standard error.

**Table 2:** Three-class solution conditional probabilities and mean (N = 4944).

	Class 1	Class 2	Class 3
	Bicultural	Low Acculturated	Non- Integrated
	765	4132	47
	15%	84%	1%
**Mean BMI**	26.1	25.8	51.7
**Language used with friends**			
Only/Mostly Spanish	0.093	0.94	0.882
Spanish and English Equally	0.479	0.049	0
Only/Mostly English	0.428	0.01	0.118
**Language used to think**			
Only/Mostly Spanish	0.18	0.976	0.873
Spanish and English Equally	0.474	0.021	0
Only/Mostly English	0.346	0.003	0.127
**Language used at home**			
Only/Mostly Spanish	0.334	0.984	0.897
Spanish and English Equally	0.353	0.01	0
Only/Mostly English	0.313	0.007	0.103
**Language used as a child**			
Only/Mostly Spanish	0.775	0.995	0.897
Spanish and English Equally	0.165	0.005	0
Only/Mostly English	0.06	0	0.103
**Language read and spoken**			
Only/Mostly Spanish	0.219	0.983	0.848
Spanish and English Equally	0.562	0.017	0
Only/Mostly English	0.219	0	0.152
**Time living in the US**			
Less than a year	0.03	0.082	0.087
1 to 4.9 years	0.084	0.223	0.13
5 to 9.9 years	0.111	0.193	0.167
10 or more years	0.776	0.502	0.615

Notes: BMI = body mass index, US = United States. Conditional probabilities that indicate likelihood within the profile are displayed in cells with white to red gradient, where white is closer to 0 and red to 1.

**Table 3:** Multinomial logistic regression using Class 1 as reference.

	Class 2			Class 3		
		95% CI			95% CI	
	OR	Lower	Upper	OR	Lower	Upper
Female	**1.45**	**1.09**	**1.92**	2.88	0.86	9.66
US Citizenship	**2.29**	**1.57**	**3.34**	1.35	0.46	3.93
Perceived overweight	**0.62**	**0.43**	**0.91**	1.82	0.49	6.73

Notes: OR = odds ratio, CI = confidence interval.

## Data Availability

The authors do not have full control of the data; the data can be obtained from (https://www.cdc.gov/nchs/nhanes/index.htm accessed on 5 March 2022).
